# Changes in ideal cardiovascular health status and risk of new-onset type 2 diabetes: The Kailuan prospective study

**DOI:** 10.1097/MD.0000000000004571

**Published:** 2016-08-26

**Authors:** Xiaoxue Liu, Jihong Shi, Anxin Wang, Qiaofeng Song, Zhe Huang, Chenrui Zhu, Xin Du, Ying Zhang, Shuohua Chen, Xizhu Wang, Shouling Wu

**Affiliations:** a Department of Cardiology, Tangshan People's Hospital; b Department of Cardiology, Kailuan Hospital, North China University of Science and Technology, Tangshan; c Department of Neurology, Beijing Tiantan Hospital; d Department of Epidemiology and Health Statistics, School of Public Health, Capital Medical University, Beijing; e Department of Ultrasonography, Hospital Affiliated to North China University of Science and Technology; f Department of Health Care Center, Kailuan Hospital, North China University of Science and Technology, Tangshan, China.

**Keywords:** cardiovascular health status, diabetes, hypertension, improvement, prospective study

## Abstract

The aim of the present study was to investigate the association between the altered ideal cardiovascular health status (ΔCHS) and the risk of developing diabetes mellitus in the Kailuan population of China.

We included 50,656 Chinese adults aged 18 years or older (11,704 men and 38,952 women) without baseline diabetes mellitus in this study. Information about 7 individual components of the cardiovascular health metrics during 2006 to 2008 was collected. A ΔCHS score was defined as the changes of ideal cardiovascular health status (CHS) from the year 2006 to 2008. New-onset diabetes was identified based on the history of diabetes, currently treated with insulin or oral hypoglycemic agents, or having a fasting blood glucose concentration ≥7.0 mmol/L during the 2010 to 2011 and 2012 to 2013 surveys. After a mean follow-up period of 3.80 years, a total of 3071 (6.06%) participants developed diabetes mellitus. Cox proportional hazards regression was used to calculate the hazard ratios and 95% confidence intervals for the CHS change and new-onset diabetes.

A strong inverse association between the positive CHS changes and lower risks of developing diabetes mellitus was observed. After adjusting for age, sex, alcohol consumption, and other potential confounders, the hazard ratios for new-onset diabetes were 0.73, 0.59, 0.49, and 0.42 (95% confidence interval: 0.37–0.82; *P* trend <0.001) for those who met ΔCHS = −1, 0, 1, and ≥2, respectively, compared with the participants with ΔCHS ≤−2.

The study concluded that the improved CHS was associated with the reduced risk of developing diabetes mellitus in this investigated Chinese population.

## 1 Introduction

Diabetes mellitus is a major risk factor for cardiovascular disease (CVD), which is the most common cause of death among adults with diabetes mellitus.^[1]^ Worldwide, an estimated 387 million adults are living with diabetes, and this number is projected to increase to 592 million by 2035.^[2,3]^ In China, a national survey during 2007 to 2008 showed that there were 92.4 million adults with diabetes and 148.2 million adults with prediabetes.^[2]^ Diabetes mellitus has become one of the greatest public health burdens and the most common cause of CVD. About 80% of patients with type 2 diabetes mellitus will develop macrovascular disease. This represents a great expenditure for health care with reduced life expectancy and quality of life.^[4,5]^ Recent studies have demonstrated that type 2 diabetes can be prevented and delayed by lifestyle modification.^[6–11]^ Prevention of diabetes mellitus will be effective on the CVD control.

The American Heart Association (AHA) has defined 7 behaviors and risk factors (smoking status, body mass index [BMI], physical activity, healthy dietary score, total cholesterol [TC], blood pressure [BP], and fasting blood glucose [FBG]) as cardiovascular health metrics and created 3 stages for each metric to reflect poor, intermediate, and ideal cardiovascular health status (CHS).^[12]^ It emphasizes the initial occurrence of risk factors and encourages individuals to adopt healthier behaviors to prevent the development of a given disease, including diabetes mellitus.

Extensive evidence has indicated the potential impact of ideal CHS on CVD, cancer, all-cause mortality,^[13–17]^ and stroke.^[18–21]^ Indeed, improvement of lifestyle has a significant protective effect from diabetes mellitus.^[6–11]^ However, fewer components or risk factors were used as continuous variables with no specific classification in those studies. The ideal cardiovascular health is defined by the AHA as the presence of both ideal health behaviors and ideal health factors.^[12]^ Absence of diabetes mellitus is 1 of the 4 favorable health factors. To improve the outcomes of the primordial prevention of diabetes and reach the 2020 impact goals of the AHA, it is important to know the impact of changes in CHS on diabetes mellitus.

In this study, we investigated whether the change of CHS (as defined by the AHA) is also significantly inversely associated with diabetes mellitus in a large population-based cohort in China.

## 2 Methods

### 2.1 Study design and subjects

**Figure 1:**
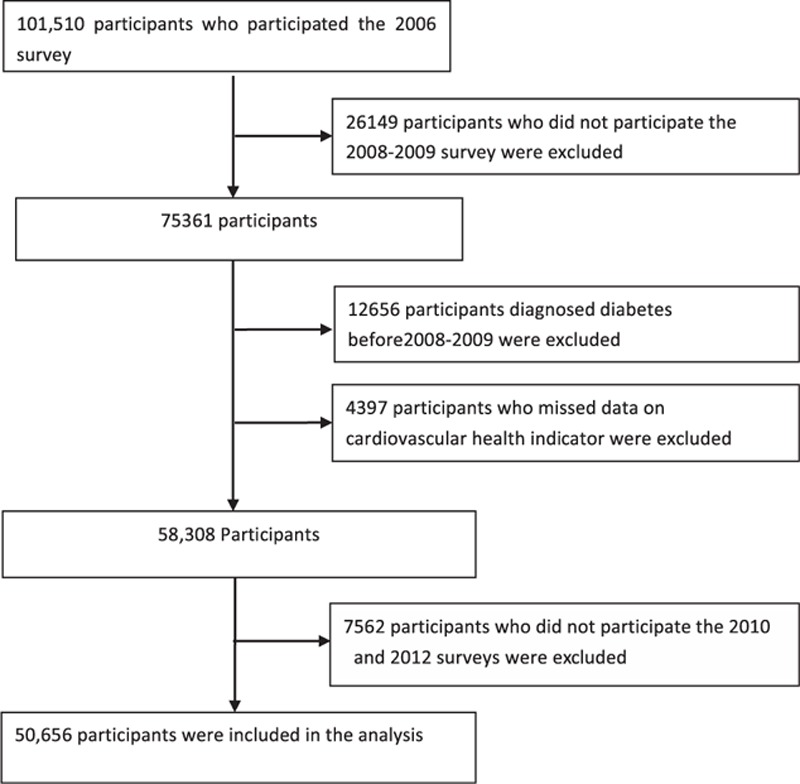
Selection of Kailuan study participants for analysis.

The Kailuan study^[15]^ was a prospective cohort study conducted in the community of Kailuan in Tangshan, which is an industrial and modern city located in the central section of the circulating Bohai Sea Gulf region of China. From June 2006 to October 2007, a total of 101,510 participants (81,110 men and 20,400 women, 18–98 years of age) were recruited to participate in the Kailuan study. At the baseline analysis, a total of 50,954 participants were excluded from the recruited population, including 33,711 participants lacking face-to-face follow-up data during the years 2008 to 2009, 2010 to 2011, or 2012 to 2013; 12,656 participants with prediagnosed diabetes mellitus until 2008 to 2009 survey; and 4397 participants without complete data regarding cardiovascular health metrics. The remaining 50,656 participants without diabetes were included in the final analysis. We considered the 2008 survey as the starting point and 2012 survey as the end point of the follow-up (Fig. 1). The follow-up evaluations included biennial measurement of laboratory parameters and recording of adverse events. The study was approved by the Ethics Committees of Kailuan General Hospital, following the guidelines outlined by the Helsinki Declaration. All participants agreed to participate in the study and provided written informed consent.

### 2.2 Assessment of cardiovascular health metrics

Information on smoking, salt intake, and physical activity was collected via questionnaires. Participants who had never smoked were defined as showing evidence of ideal health. Former smokers were defined as showing evidence of intermediate health. Participants who were currently smoking were defined as showing evidence of poor health. Self-reported salt intake (individual levels) was classified as “low,” “medium,” or “high.” However, there is no information on the amount of salt consumed (g/d). “Low” salt intake was defined as a surrogate of ideal dietary behavior, consistent with current guideline recommendations and because salted food intake is a serious issue in China. Likewise, “medium” and “high” salt intakes were defined as intermediate and poor diet behaviors, respectively. Physical activity was evaluated from responses to questions about the type and frequency of physical activity at work and during leisure time and permits. The ideal, intermediate, and poor physical activities were defined as ≥80, 1 to 79, and 0 minutes of moderate or vigorous activity per week, respectively.^[18]^

Height was measured to an accuracy of 0.1 cm using a tape measure, and weight was measured to the nearest 0.1 kg with calibrated platform scales. Body weight (kg) was divided by the square of height (m^2^) to determine the BMI. BMI was calculated as body weight (kg) divided by the square of height (m^2^). BP was measured to the nearest 2 mm Hg with a mercury sphygmomanometer following the standard procedures. Three readings of systolic blood pressure (SBP) and diastolic blood pressure (DBP) were taken at a 5-minute interval after participants had rested in a chair for at least 5 minutes. The average of the 3 readings was used for data analysis. A 10-second and 12-lead electrocardiography was used to measure the resting heart rate (RHR) after the individual had rested in the supine position for 5 minutes. The number of R–R intervals (number of QRS complexes, 1) was divided by the time between the first and the last beat, and the results were converted to beats per minute.^[22]^

Blood samples were collected from the antecubital vein after overnight fasting. Blood was collected in vacuum tubes containing ethylene diamine tetraacetic acid (EDTA), and samples were centrifuged at 300 × *g* for 10 minutes at room temperature to obtain plasma. FBG was measured using the hexokinase/glucose-6-phosphate dehydrogenase method.^[23]^ TC and triglycerides were measured enzymatically. High-density lipoprotein cholesterol and low-density lipoprotein cholesterol levels were measured using a direct test method^[24]^ (interassay coefficient of variation <10%; Mind Bioengineering Co Ltd, Shanghai, China). High sensitive C-reactive protein (hs-CRP) was measured by high-sensitivity nephelometry assay (Cias Latex CRP-H; Kanto Chemical, Tokyo, Japan). Serum uric acid (UA) concentrations were determined using an oxidase method. All biochemical variables were measured at the central laboratory of Kailuan General Hospital using a Hitachi autoanalyzer (Hitachi 747; Hitachi, Tokyo, Japan).

According to the AHA definitions,^[12]^ ideal BMI was defined as BMI <25 kg/m^2^, intermediate BMI as BMI 25 to 29.9 kg/m^2^, or poor BMI as BMI ≥30 kg/m^2^. Ideal BP was defined as SBP <120 mm Hg and DBP <80 mm Hg; intermediate BP as 120 ≤ SBP ≤ 139 mm Hg, 80 ≤ DBP ≤ 89 mm Hg, or treated to the goal; and poor BP as SBP ≥140 mm Hg, DBP ≥90 mm Hg, or treated to SBP/DBP >120/80 mm Hg. Ideal FBG was defined as <100 mg/dL, intermediate blood glucose as 100 to 125 mg/dL or treated to <100 mg/dL, and poor as blood FBG level ≥126 mg/dL or treated to ≥100 mg/dL. The TC status was classified as ideal with <200 mg/dL for the untreated, intermediate with 200 to 239 mg/dL or treated to <200 mg/dL, and poor with ≥240 mg/dL or treated to ≥200 mg/dL.

### 2.3 Assessment of potential covariates

The demographic and clinical characteristics, including age, sex, alcohol use, personal monthly income, education, history of diseases, and family history of diseases, were collected via questionnaires. Age was classified into 2 categories: <60 and ≥60 years old. Previous history of diseases, including myocardial infarction, stroke, and cancer, was recorded based on self-report. The use of antihypertensive, cholesterol-lowering, and glucose-lowering medications within the past 2 weeks before the baseline interview was collected based on self-reported. The average monthly income was categorized as “<¥600,” “¥600 to 800,” or “≥¥800.” The educational attainment was categorized as “illiteracy or primary,” “middle school,” and “high school or above.”

### 2.4 Assessment of new-onset diabetes

In line with the American Diabetes Association guidelines, participants were identified as having diabetes mellitus if they were currently treated with insulin or oral hypoglycemic agents, or had a FBG concentration ≥7.0 mmol/L in the 2010 to 2011 and 2012 to 2013 surveys.^[25]^

### 2.5 Statistical analyses

Continuous variables were described as means and were compared using analyses of variance or the Kruskal–Wallis test. Categorical variables were described as percentages and were compared using χ^2^ tests. To examine the cumulative effects of 7 cardiovascular health metrics, we created a dichotomized variable for each component of the health metrics: “ideal” was coded as 2, “intermediate” was coded as 1, and “poor” was coded as 0. The total ideal CHS score of each individual ranged from 0 to 14. CHS changes were calculated by subtracting the total score for the metrics obtained in 2006 from the total score obtained in 2008. Participants were divided into 5 categories based on the quintiles of CHS changes. Person-years were calculated from the date when the 2008 interview was conducted to the date when diabetes was detected (depending on the analysis in question), date of death, or date of participating in the last interview in this analysis, whichever came first.

Cox proportional hazards regression was used to estimate the risk of diabetes by calculating the hazard ratios (HRs) and 95% confidence intervals (CIs). We fitted 3 multivariate proportional hazards models. Model 1 adjusted for age, sex, and cardiovascular health scores in 2006. Model 2 further adjusted for education level, income level, and drinking. Model 3 further adjusted for hs-CRP, UA, RHR in 2006, and family history of diabetes and myocardial infarction. Because 11 hospitals participated in the study, we used a Cox proportional hazards model with a sandwich covariance matrix as a random effect to account for the potentially confounding effect of multiple hospitals participating in the study. All interactions were analyzed by multivariate Cox proportional hazards modeling. Statistical analyses were performed using SAS 9.3 (SAS Institute, Cary, NC). All statistical tests were 2-sided, and the significance level was set at 0.05.

## 3 Results

Of the 101,510 Kailuan study participants, a total of 50,656 eligible participants (23.10% women) were analyzed in our study. We divided the participants into 5 categories according to the changes in CHS from the year 2006 to 2008. Overall, there were 20.5% participants with the altered ideal cardiovascular health status (ΔCHS) ≤−2, 20.4% participants with ΔCHS = −1, 24.9% participants with ΔCHS = 0, 18.7% participants with ΔCHS = 1, and 15.6% participants with ΔCHS ≥2. The average age of the remaining population was 48.65 years. After a mean follow-up period of 3.8 years, a total of 3071 (6.06%) participants developed diabetes mellitus. We compared the baseline characteristics (2006 data) of the 5 categories of participants as summarized in Table 1. Comparing the participants with ΔCHS ≤−2, the other groups had a lower proportion of men, higher educational levels, and higher incomes.

**Table 1:**
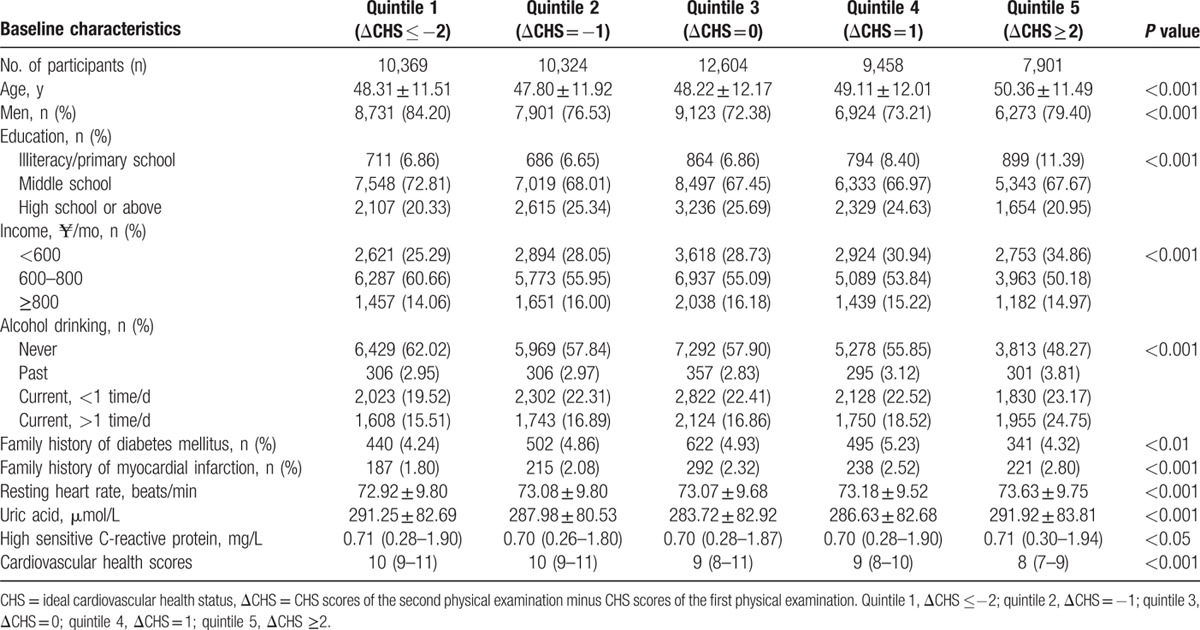
Characteristics in 2006 according to the change of cardiovascular health scores from 2006 to 2008.

The association between the changes in cardiovascular health scores and the incidence of diabetes mellitus is shown in Table 2. We adjusted the association for sex, age, education, income, drinking, hs-CRP, UA, RHR, family history of diabetes and myocardial infarction, and cardiovascular health scores at baseline. The participants with a ΔCHS value ≥2 had reduced incidence of diabetes mellitus (adjusted HR: 0.42, 95% CI: 0.37–0.48), compared with participants with ΔCHS ≤−2. We also found that the risk of diabetes decreased by approximately 16% as ΔCHS increased by a score of 1 point (HR: 0.84, 95% CI: 0.82–0.86).

**Table 2:**
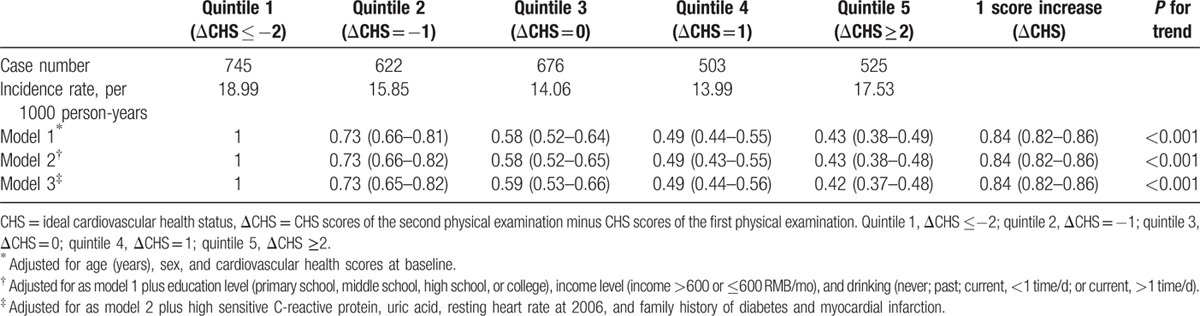
Hazard ratios and 95% confidence intervals of diabetes according to change of cardiovascular health scores.

We divided the participants into different groups according to their age, sex, and baseline cardiovascular health scores and calculated the HR for the incidence of diabetes, adjusted for age, sex, baseline cardiovascular health scores, education, income, alcohol intake, RHR, and family history of diabetes and myocardial infarction. As shown in Table 3, the risk of new-onset diabetes for the participants with ΔCHS ≥2 (fifth quintile) decreased by 68% for females (HR: 0.32, 95% CI: 0.23–0.43) and 54% for males (HR: 0.46, 95% CI: 0.40–0.53), when compared with the participants with ΔCHS ≤2. The risk of diabetes for the participants aged <60 years and those >60 years old was reduced 56% (HR: 0.44, 95% CI: 0.38–0.51) and 65% (HR: 0.35, 95% CI: 0.26–0.48), respectively. The risk of diabetes for the participants whose baseline cardiovascular health scores were ≤9 and >9 was decreased 56% (HR: 0.44, 95% CI: 0.38–0.51) and 59% (HR: 0.41, 95% CI: 0.28–0.58), respectively. The significant inverse associations of risk of diabetes with the changes in cardiovascular health scores were found in the age group (*P* trend <0.001), males (*P* trend <0.001), females (*P* trend <0.001), baseline CHS ≤9 (*P* trend <0.001), or baseline CHS >9 (*P* trend <0.001). There were no interactions between the changes in cardiovascular health scores with age (*P* = 0.99), sex (*P* = 0.12), or baseline CHS (*P* = 0.58).

**Table 3:**
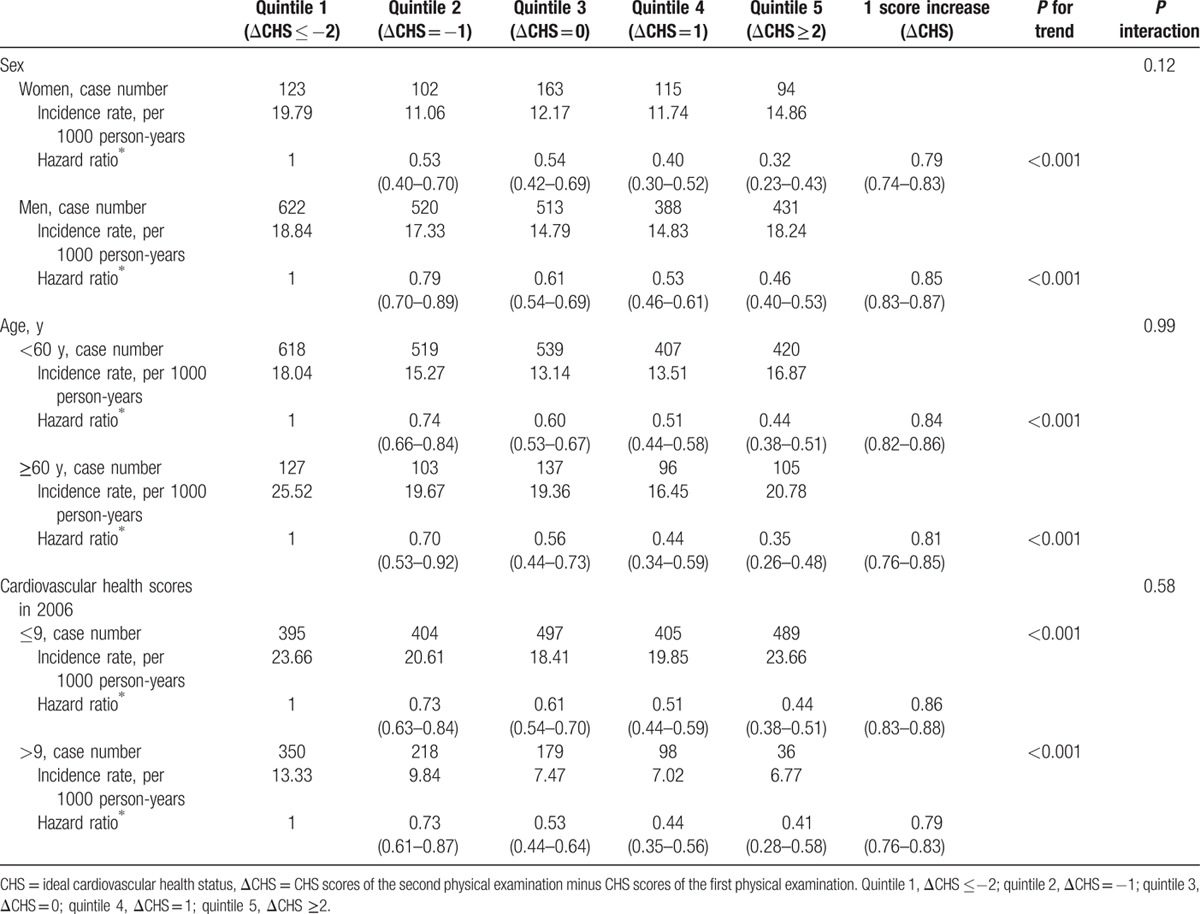
Incidence of diabetes in different groups according to the change of cardiovascular health scores.

To determine the impact of any single cardiovascular health metrics on the associations between the combined CHS change and incidence of diabetes, we removed 1 out of 7 cardiovascular health metrics at a time and reexamined these associations (Table 4). The changes in the remaining cardiovascular health metrics were still significantly associated with the incidence of diabetes.

**Table 4:**
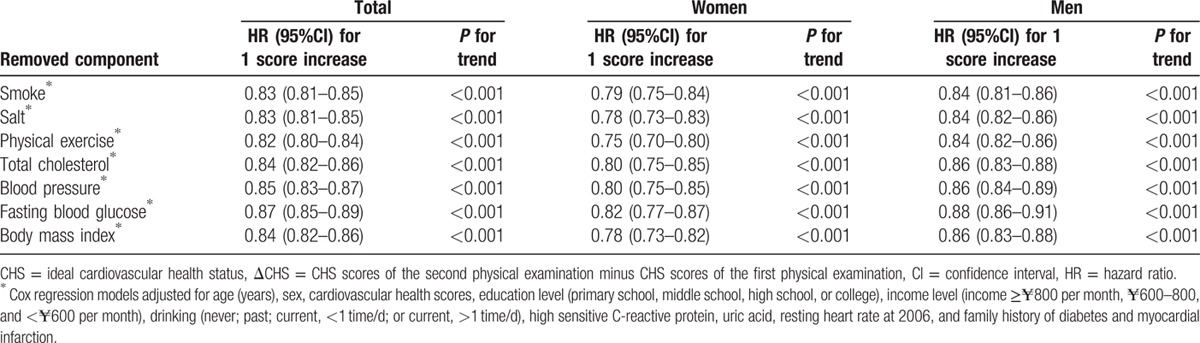
HRs and 95% CIs of diabetes according to change of cardiovascular health scores from 2006 to 2008, after 1 individual cardiovascular health is removed from the total score.

Table 5 compares the baseline characteristics of those included in the analysis with those who did not participate in the 2010 and 2012 surveys. Compared with the group of participants, the group of nonparticipants was much older, dominated by men, and current alcohol consumers, and participants with a higher RHR, UA, and high-sensitive C-reactive protein, lower education level, and lower reported income (all *P* < 0.001).

**Table 5:**
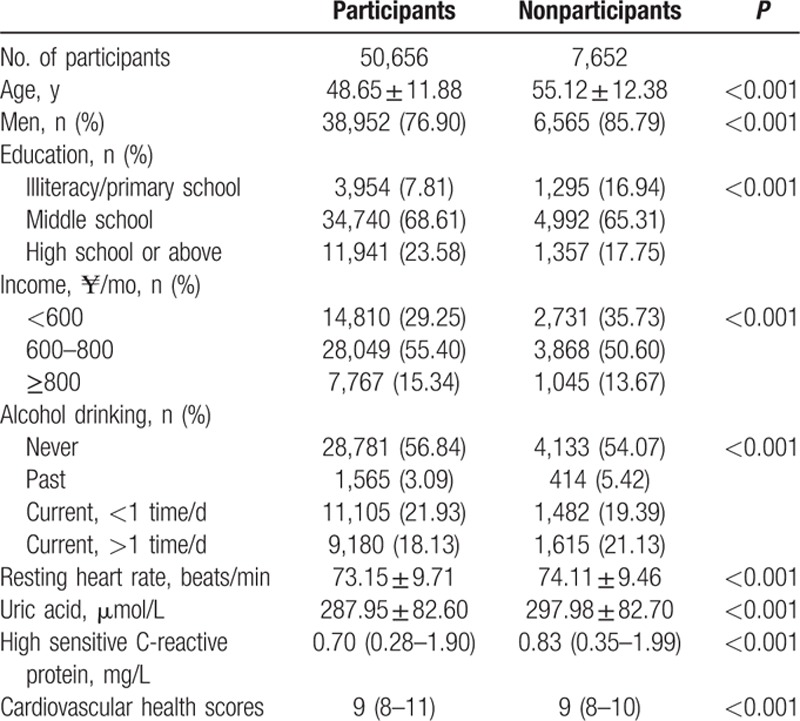
Clinical characteristics in 2006 according to the participants who participated or not participated in the follow-up in 2010 and 2012 surveys.

## 4 Discussion

In this large prospective cohort study of 50,656 participants, we analyzed the relationship between cardiovascular health and diabetes. We observed a significant association of the changes in the ideal cardiovascular health index with the incidence of diabetes mellitus in the general Chinese population. This relationship was sustained after being adjusted for age, sex, baseline cardiovascular health scores, education, income, alcohol intake, RHR, and family history of diabetes and myocardial infarction. Our results indicate that a healthy lifestyle may reduce the risk of diabetes.

The results reported by Eriksson and Lindgarde show that type 2 diabetes mellitus can be prevented by controlling diet and increasing physical exercise in the Sweden population.^[8]^ Similar results were obtained by Pan et al in a Chinese population.^[26]^ Another study also indicated that combined diet control and physical activity promotion programs are effective at reducing diabetes incidence and improving cardiometabolic risk factors.^[9]^ Moreover, age, BMI, and lifestyle factors are well-established risk factors for the development of diabetes in many of epidemiological evidences.^[10,11,27]^ In the above-mentioned studies, however, a few components were applied or the risk factors were used as continuous variables without specific classification. Indeed, using a single index or 1-time sampling data appeared to be not enough for obtaining accurate and credible results. Our study has taken the time-associated changes in multiple indexes (7 of cardiovascular health metrics) into consideration, which may improve the accuracy and reliability of our study.

We found an inverse association between the CHS change and diabetes. In addition, to further explore the association between the CHS changes and diabetes, we investigated the association stratified by sex and age. Remarkably, we found that the association between the CHS change and diabetes remained significant in subgroups based on the index at baseline after being adjusted for education level, income level, drinking, hs-CRP,^[28,29]^ UA,^[30,31]^ and RHR^[23]^ in 2006. This association supports the hypothesis that the improvement in an individual's CHS has an independent favorable effect on reducing the risk of diabetes. At the same time, it is encouraging to note that people with poor health transited into intermediate or ideal health by improving their health lifestyle.

The baseline ideal cardiovascular health score plays an important role in the development of cardiometabolic outcome. An ideal CHS in the early stage of life may contribute to the health status in the later life. Therefore, we explored the association between the CHS change and diabetes in participants with baseline CHS <9 or >9 points separately. The improvement of CHS reduced the incidence of diabetes persistently. These findings suggest that the changes in the CHS in the populations with poor or ideal health at baseline continue to have an important influence on the later health outcomes.

Pan et al found whether a change in diet was more efficient than that in exercise in prevention of diabetes, or vice visa.^[26]^ In our study, we did not analyze these changes separately. However, we focused on the changes in lifestyle that were as extensive as possible for each subject. Therefore, we conducted additional analyses removing each ideal health metric at a time and repeated our analyses individually. Although the strength of the association between the changes in the remaining CHS and incident diabetes was attenuated after a single component of ideal cardiovascular health metrics was removed from the total score, the association remained statistically significant. These results indicate that all of the ideal health metrics are equally important, suggesting that the improvement of total CHS can be used as an alternative method to prevent becoming diabetic.

Our study has several strengths. This is the first prospective study to address the association between CHS changes and the incidence of diabetes mellitus in both Chinese men and women. Other strengths of our study included large sample size and availability of several important potential confounders such as RHR, blood concentrations of hs-CRP, UA, and family history of diabetes and myocardial infarction. However, some limitations in our results should also be noticed. First, it is a single-center cohort study; all participants were living in a society associated with the Kailuan Coal Company. The participants were not nationally representative. Our findings can therefore not directly be generalized to other Chinese populations with different regional backgrounds. However, studying such a geographically confined and controlled population has greatly reduced residual confounding due to diverse socioeconomic factors and lifestyle patterns. Second, the diagnosis of diabetes was based on a single measure of FBG without using oral glucose tolerance test, which is due to lack of availability of oral glucose tolerance test data in such a large cohort. Third, a great number of participants were excluded due to the lack of follow-up data from 2010 to 2013, which may generate bias results of the statistical analysis. After comparing participants and nonparticipants, we found that the majority of the nonparticipants were aged and had higher levels of hs-CRP, RHR, UA, current drinking, and lower education level, which are risk factors of diabetes. Finally, information on smoking, salt intake, and physical activity was collected via questionnaires, which could have underestimated the results. However, specially trained doctors and nurses performed all measurements, using standard protocols, which was in accordance with our previous articles.^[15,18,32,33]^

In conclusion, our findings provide further evidence that the improvement of CHS is associated with a reduced risk of diabetes in this investigated Chinese population.
